# Oligomerization Requirements for MX2-Mediated Suppression of HIV-1 Infection

**DOI:** 10.1128/JVI.02247-15

**Published:** 2015-12-17

**Authors:** Matthew D. J. Dicks, Caroline Goujon, Darja Pollpeter, Gilberto Betancor, Luis Apolonia, Julien R. C. Bergeron, Michael H. Malim

**Affiliations:** a King's College London, Department of Infectious Diseases, London, United Kingdom; b Centre d'Études d'Agents Pathogènes et Biotechnologies pour la Santé (CPBS), Montpellier, France; c University of British Columbia, Department of Biochemistry and Molecular Biology, Vancouver, Canada

## Abstract

Human myxovirus resistance 2 (MX2/MXB) is an interferon-stimulated gene (ISG) and was recently identified as a late postentry suppressor of human immunodeficiency virus type 1 (HIV-1) infection, inhibiting the nuclear accumulation of viral cDNAs. Although the HIV-1 capsid (CA) protein is believed to be the viral determinant of MX2-mediated inhibition, the precise mechanism of antiviral action remains unclear. The MX family of dynamin-like GTPases also includes MX1/MXA, a well-studied inhibitor of a range of RNA and DNA viruses, including influenza A virus (FLUAV) and hepatitis B virus but not retroviruses. MX1 and MX2 are closely related and share similar domain architectures and structures. However, MX2 possesses an extended N terminus that is essential for antiviral function and confers anti-HIV-1 activity on MX1 [MX1(N_MX2_)]. Higher-order oligomerization is required for the antiviral activity of MX1 against FLUAV, with current models proposing that MX1 forms ring structures that constrict around viral nucleoprotein complexes. Here, we performed structure-function studies to investigate the requirements for oligomerization of both MX2 and chimeric MX1(N_MX2_) for the inhibition of HIV-1 infection. The oligomerization state of mutated proteins with amino acid substitutions at multiple putative oligomerization interfaces was assessed using a combination of covalent cross-linking and coimmunoprecipitation. We show that while monomeric MX2 and MX1(N_MX2_) mutants are not antiviral, higher-order oligomerization does not appear to be required for full antiviral activity of either protein. We propose that lower-order oligomerization of MX2 is sufficient for the effective inhibition of HIV-1.

**IMPORTANCE** Interferon plays an important role in the control of virus replication during acute infection *in vivo*. Recently, cultured cell experiments identified human MX2 as a key effector in the interferon-mediated postentry block to HIV-1 infection. MX2 is a member of a family of large dynamin-like GTPases that includes MX1/MXA, a closely related interferon-inducible inhibitor of several viruses, including FLUAV, but not HIV-1. MX GTPases form higher-order oligomeric structures, and the oligomerization of MX1 is required for inhibitory activity against many of its viral targets. Through structure-function studies, we report that monomeric mutants of MX2 do not inhibit HIV-1. However, in contrast to MX1, oligomerization beyond dimer assembly does not seem to be required for the antiviral activity of MX2, implying that fundamental differences exist between the antiviral mechanisms employed by these closely related proteins.

## INTRODUCTION

Type 1 interferons (IFNs) are key cytokine mediators of innate immunity and promote an antiviral state in response to acute viral infection through the upregulation of interferon-stimulated genes (ISGs) (1, 2). Human myxovirus resistance 2 (MX2/MXB) recently has been identified as an interferon-inducible late postentry inhibitor of human immunodeficiency virus type 1 (HIV-1) infection (3–5). MX2 imposes a block to viral replication that occurs after reverse transcription but prior to nuclear import, preventing the accumulation of nascent viral cDNAs in the nucleus (3, 4). The HIV-1 capsid (CA) protein is believed to be the viral determinant of inhibition (3–5); indeed, MX2 has been shown to interact with recombinant capsid-nucleocapsid nanotubes *in vitro*. However, the precise mechanism of antiviral action remains unclear, not least because point mutations in CA that permit viral escape from MX2-mediated suppression do not appear to inhibit binding of MX2 (6–8).

Human MX2 is a member of a family of large dynamin-like GTPases that includes human MX1/MXA, a long-established interferon-induced inhibitor of a broad range of RNA and DNA viruses (9). Viruses inhibited by MX1 include influenza A virus (FLUAV), La Crosse virus, Thogoto virus, measles virus, and hepatitis B virus but not retroviruses such as HIV-1 (3, 9). While the precise mechanism of action may differ for different viruses, GTPase activity and oligomerization are required for antiviral activity of MX1 against FLUAV, the prototypical target of MX1 restriction (10–12). Current models propose that MX1 forms oligomeric rings that interact with viral nucleoprotein complexes (13–15) and constrict around their target upon GTP hydrolysis (9, 16, 17). In contrast, GTPase activity is not essential for MX2 function (3, 4, 18, 19), and the importance of oligomerization beyond dimer formation for antiviral activity has been questioned (6, 20), pointing to substantial differences in mechanisms of action.

**FIG 1:**
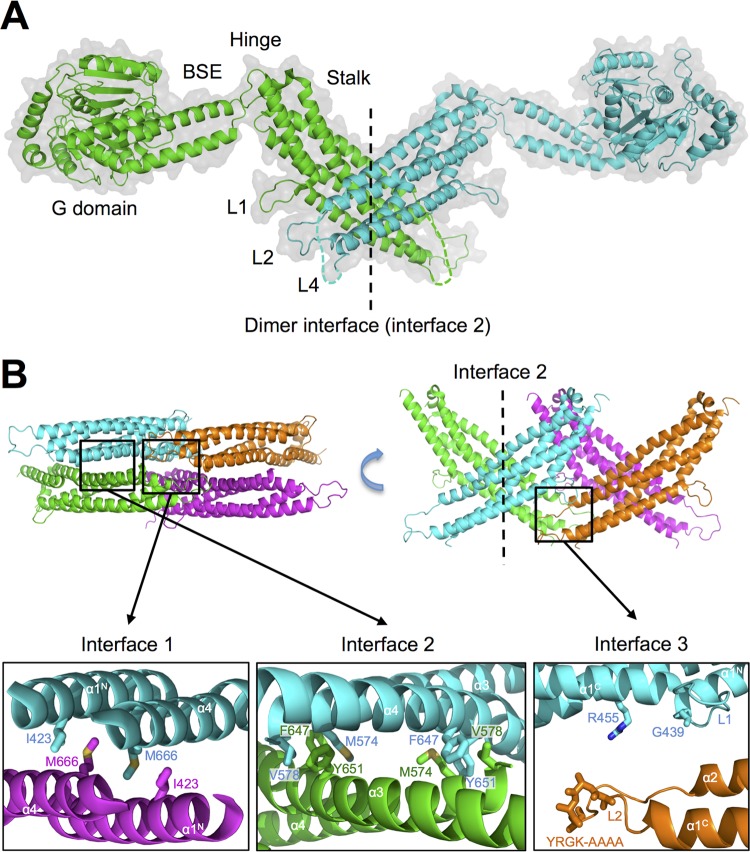
Structure and predicted oligomerization interfaces of human MX2. (A) Crystal structure of the MX2 dimer (protomers in green and blue) from Fribourgh et al. (6) (PDB entry 4WHJ), in cartoon representation. The G domain, bundle signaling element (BSE), hinge region, stalk domain, and L1, L2, and L4 loops are indicated. The dimer interface (interface 2) between stalk domains is also shown (dashed line). (B) Predicted oligomerization interfaces of MX2. A dimer of stalk dimers (green and blue; purple and orange) is shown with dimer interface 2 and putative oligomerization interfaces 1 and 3 indicated. The latter two interfaces correspond to crystallographic symmetry-related interfaces. Residues predicted to stabilize each interface by homology to MX1 are shown as sticks. All figures were generated using the PyMol Molecular Graphics System (Schrödinger).

Human MX1 and MX2 share 63% sequence identity and the same domain architecture, and the structures of the two proteins are very similar, with a root-mean-square deviation of 6.4 Å for backbone atoms (6, 16, 21). Both proteins comprise an amino-terminal GTPase domain (G domain) and a carboxy-terminal stalk domain that are connected by a tripartite bundle signaling element (BSE) (Fig. 1). Comprehensive structural and biochemical studies with MX1 have shown that the stalk domain is critical for oligomerization (10, 17), while the BSE transmits conformational changes between the G domain and stalk upon GTP binding and hydrolysis (16).

MX2 possesses an extended N terminus compared to that of MX1; indeed, this region has been shown to be essential for anti-HIV-1 activity (22, 23). MX2 also exists as two isoforms (24) due to the presence of an alternative initiation methionine codon at position 26. The longer 78-kDa form is antiviral and is partly associated with the nuclear envelope, while the shorter 76-kDa isoform is not antiviral and is cytoplasmic owing to the absence of a nuclear envelope targeting sequence. The precise role of the MX2 N-terminal region presently is unclear, but it does contain essential functional determinants other than a nuclear envelope targeting sequence (22, 23, 25). More specifically, a triple-arginine motif at positions 11 to 13 is required for anti-HIV-1 activity (20) and recently has been proposed as an HIV-1 CA binding motif (26). Furthermore, the N-terminal 91 amino acids of MX2 are sufficient to confer anti-HIV-1 activity on MX1 [MX1(N_MX2_)] and, remarkably, on heterologous protein scaffolds (20, 22, 23, 25). Importantly, chimeric MX1(N_MX2_) recapitulates many features and specificities of MX2-mediated antiviral activity (23); therefore, it serves as a valuable tool in dissecting its mechanism of action.

Here, we performed site-directed mutagenesis studies to investigate in detail the importance of oligomerization for the inhibition of HIV-1 infection in the context of both human MX2 and the MX1(N_MX2_) chimera. Basing our work on previous studies performed with MX1 and FLUAV (10, 16) and recently published structural data for an amino-terminally truncated form of MX2 (6), we generated a series of point mutants at the various oligomerization interfaces within the stalk domain and determined their anti-HIV-1 activity (Table 1). Protein cross-linking and coimmunoprecipitation assays were used to assess the oligomerization state of the mutant proteins. We show that monomeric forms of MX2 and MX1(N_MX2_) lack antiviral function, but in contrast to MX1 inhibition of FLUAV, oligomerization beyond dimer assembly does not appear to be required for anti-HIV-1 activity.

**TABLE 1:** Summary of oligomerization mutants used in this study^*a*^

MX1 mutant	Region	MX1 oligomerization	MX1 antiviral activity^*b*^ (FLUAV)	Corresponding MX2 mutation
WT		Tetramer	Yes	
L617D	Stalk interface 1	Dimer	No	M666D
I376D	Stalk interface 1	Dimer	—	I423D
M527D	Stalk interface 2	Monomer	No	M574D
F602D	Stalk interface 2	Monomer	—	Y651D
YRGR_440_-AAAA_443_	Stalk interface 3	Dimer	—	YRGK_487_-AAAA_490_
R408D	Stalk interface 3	Dimer	No	R455D
G392D	Stalk interface 3	Dimer	—	G439D
E632A	BSE hinge	Dimer	Partial	E681A
R640A	BSE hinge	Dimer	No	R689A
ΔL4	ΔL4 loop	Dimer	No	ΔL4

a Shown is an overview of mutations previously shown to disrupt MX1 oligomerization (10, 16), their location within MX1, oligomerization state in sedimentation equilibrium assays with recombinant protein (10, 16), and consequences for anti-FLUAV activity (10, 16). Mutations generated in MX2 for this study, corresponding to those previously characterized for MX1, are shown. The same MX1 mutations shown here also were introduced in the context of the MX1(N_MX2_) chimera in this study.

b A dash indicates that the anti-FLUAV activity of the respective mutant was not tested.

## MATERIALS AND METHODS

### Cell culture and plasmid constructs.

Human 293T cells and U87-MG CD4^+^ CXCR4^+^ (3) cells were cultured in Dulbecco's modified Eagle medium (DMEM) supplemented with fetal bovine serum (10%), l-glutamine, and penicillin-streptomycin. Site-directed mutagenesis was performed on human *MX2* or *MX1*(*N_MX2_*) (residues 1 to 91 of MX2 appended to residues 44 to 662 of human MX1 [23]) constructs containing a C-terminal FLAG tag using standard PCR amplification techniques. For the loop 4 (L4) deletion mutants, overlapping PCR was used to delete residues 580 to 608 of MX2, and the sequence corresponding to residues 533 to 561 of native human MX1 (10) was deleted in the context of the MX1(N_MX2_) chimera. Mutant constructs were cloned into EasiLV-MCS (3) using BamHI and XhoI restriction sites. FLAG-tagged mutants then were subcloned into pCAGGS (Addgene) using BclI and XhoI restriction sites. *GFP*, *MX1*, and *MX2* were subcloned into pCMV4.HA using Acc65I and XbaI to introduce a triple-hemagglutinin (HA) tag at the C terminus before further subcloning into EasiLV-MCS or pCAGGS. An *MX1*(*N_MX2_*) construct with a C-terminal triple-HA tag was generated by overlapping PCR using HA-tagged *MX1* as a template and cloned into pCAGGS using NotI and XhoI restriction sites.

### HIV-1 vector infectivity assays.

HIV-1 infectivity assays were performed as described previously (3, 20, 23). Briefly, U87-MG CD4^+^ CXCR4^+^ cells were transduced with C-terminally FLAG-tagged *CD8*, *MX1*, *MX2*, or *MX1*(*N_MX2_*) gene constructs using the doxycycline-inducible EasiLV lentiviral vector system (3). After 6 h, transgene expression was induced for ∼72 h prior to challenge by the addition of doxycycline [0.05 μg/ml for *MX1*(*N_MX2_*) constructs, 0.5 μg/ml for all other constructs]. EasiLV transduction efficiency typically was above 85% and was assessed by measuring the percentage of cells expressing E2-crimson (coexpressed via an internal ribosome entry site [IRES]) by flow cytometry (FACSCanto II; BD Biosciences). Protein expression was assessed by immunoblotting; cell pellets were lysed in sample buffer (200 mM Tris-HCl, pH 6.8, 5.2% SDS, 20% glycerol, 0.1% bromophenol blue, 5% β-mercaptoethanol), resolved by SDS-PAGE, and analyzed using anti-FLAG (mouse monoclonal M2; Sigma-Aldrich) and anti-Hsp90 (rabbit; Santa Cruz Biotechnology) antibodies by chemiluminescence. For viral infection, ∼2.5 × 10^4^ to 5 × 10^4^ cells were seeded in 96-well plates and challenged with a vesicular stomatitis virus G protein (VSV G)-pseudotyped 8.91 HIV-1 Gag-Pol-based cytomegalovirus (CMV) immediate early-enhanced green fluorescent protein lentiviral vector (HIV-1/GFP) at a multiplicity of infection (MOI) of 0.2. Productive infection was enumerated by flow cytometry as the percentage of E2-crimson-positive cells expressing GFP at ∼48 h postinfection by flow cytometry. EasiLV particles and challenge HIV-1/GFP vector stocks were prepared as described previously (3, 23).

### Protein cross-linking.

U87-MG CD4^+^ CXCR4^+^ cells (3) were transduced in 6-well plates with EasiLV vectors carrying *MX1*, *MX2*, or *MX1*(*N_MX2_*) FLAG-tagged constructs, and expression was induced with doxycycline as described previously. After ∼72 h, cells were harvested in phosphate-buffered saline (PBS; Life Technologies), and cell pellets were resuspended in cell lysis buffer (1× PBS, 0.5% Triton X-100, 1× protease inhibitor cocktail [Roche]) and lysed by brief sonication. Lysate was cleared by centrifugation at 1,500 × *g* for 10 min. The covalent cross-linking agent disuccinimidyl suberate (DSS; Thermo Scientific), a noncleavable, amine-reactive N-hydroxysuccinimide (NHS) ester, was dissolved in dimethyl sulfoxide (DMSO) at a stock solution of 10 mg/ml and added to cell lysates at a final concentration of 100 μg/ml or 25 μg/ml. Samples containing 1% DMSO only were included as a control. Cross-linking reaction mixtures were incubated at room temperature for 1 h before the addition of protein sample buffer and resolved by SDS-PAGE on a 6% acrylamide gel. Immunoblotting was performed using a horseradish peroxidase (HRP)-conjugated anti-FLAG antibody (mouse monoclonal M2; Sigma-Aldrich) and chemiluminescence.

For cross-linking followed by immunoprecipitation, 293T cells were cotransfected with pCAGGS carrying FLAG-tagged *MX2* or *MX1*(*N_MX2_*) and pCAGGS carrying triple-HA-tagged *MX2* or *MX1*(*N_MX2_*) using TransIT-2020 reagent (Mirus). After ∼30 h, cells were harvested and DSS cross-linking performed as described previously. After 1 h, cross-linker was quenched with 50 mM Tris-HCl for 15 min, and cross-linked protein was immunoprecipitated in modified radioimmunoprecipitation assay (RIPA) buffer (50 mM Tris-HCl, pH 7.6, 150 mM NaCl, 1% Triton X-100, 1% sodium deoxycholate, 0.5% SDS) using anti-HA magnetic beads (Pierce) for 2 h at 4°C. Beads were washed 4 times in modified RIPA buffer with the NaCl concentration raised to 500 mM prior to the addition of sample buffer, resolution by SDS-PAGE, and detection of HA- or FLAG-tagged MX2 or MX1(N_MX2_) by immunoblotting using HRP-conjugated anti-HA (rat monoclonal 3F10; Sigma-Aldrich) or anti-FLAG antibodies.

### Coimmunoprecipitation.

293T cells were seeded in 6-well plates and cotransfected with pCMV4- and pCAGGS-based plasmids encoding triple HA-tagged and FLAG-tagged constructs, respectively, using polyethylenimine. After ∼30 h, cells were harvested, resuspended in hypotonic lysis buffer (10 mM Tris-HCl, pH 8.0, 10 mM KCl, 1× protease inhibitor cocktail [Roche]), and lysed by Dounce homogenization. Lysates were cleared by centrifugation at 1,500 × *g* for 10 min, and KCl and Triton X-100 were added to cleared lysates at final concentrations of 100 mM and 0.3%, respectively. HA-tagged proteins were immunoprecipitated using anti-HA magnetic beads (Pierce) for 2 h at 4°C, and beads were washed a further 4 times in wash buffer (10 mM Tris-HCl, pH 8.0, 200 mM KCl, 0.3% Triton X-100) before the addition of sample buffer. HA- and FLAG-tagged proteins were resolved on 10% acrylamide gels by SDS-PAGE and detected by immunoblotting using HRP-conjugated anti-HA or anti-FLAG antibody.

## RESULTS

### Importance of the stalk oligomerization interfaces, BSE hinge region, and L4 loop for the antiviral activity of MX2.

The crystal structure of the human MX2 dimer (residues 84 to 715) is shown in Fig. 1A (6). Human MX2 previously has been shown to form higher-order oligomers (27), and oligomerization via the stalk domain has been predicted to proceed in a manner similar to that of the model described for human MX1 (10). Figure 1B shows two crystallographic symmetry-related MX2 stalk dimers, highlighting the interaction interface involved in dimerization (interface 2) and putative higher-order oligomerization interfaces (interfaces 1 and 3) corresponding to those described and characterized in detail for MX1 (10) and the related GTPase dynamin (28). For MX1, the dimer has been proposed as the basic structural unit (10), with interfaces 1 and 3 enabling tetramerization and subsequent formation of the higher-order ring structures that have been observed *in vitro* (13). Recombinantly expressed wild-type MX1 forms stable tetramers in solution, while proteins with mutations in stalk interface 1 or 3 are dimeric, and proteins with mutations in interface 2 are monomeric (10) (Table 1). Mutation at the BSE hinge region of MX1 (E632A and R640A) and deletion of the L4 loop (which acts as a viral specificity determinant in the context of MX1 [29, 30]) also disrupt MX1 tetramer formation (10, 16). Importantly, disruption at each of these interfaces led to the abrogation of MX1's anti-FLUAV activity (10, 16) (Table 1).

In the current study, mutations corresponding to those characterized previously for MX1 (10, 16) were generated at the predicted stalk interfaces, BSE hinge region, and L4 loop of MX2, as outlined in Table 1. The locations of targeted residues within the MX2 stalk are shown in Fig. 1B. Putative dimer interface residues V578 and F647 were identified from the MX2 crystal structure alone (6). The MX2 L2 loop mutation YRGK_487_-AAAA_490_, corresponding to YRGR_440_-AAAA_443_ in MX1, facilitated crystallization of both proteins (6, 10) and previously has been shown to disrupt stalk interface 3 in the context of MX1 (10).

**FIG 2:**
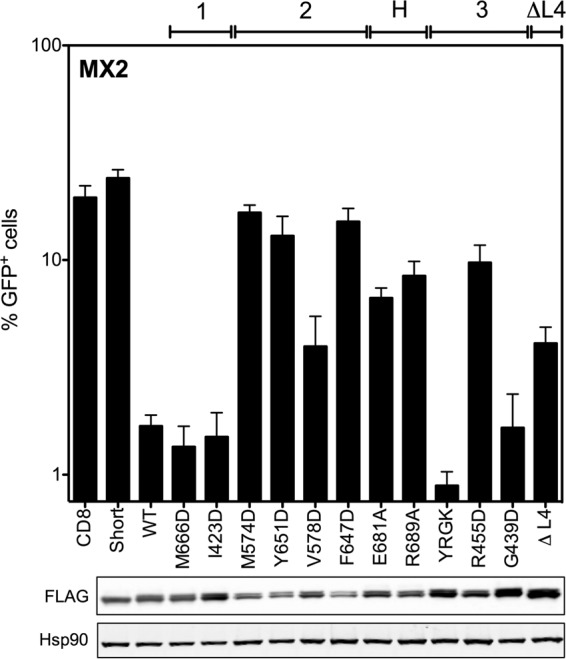
Mutations at the dimer interface, oligomerization interface 3, and hinge region impair anti-HIV-1 activity of MX2. (Upper) U87-MG CD4^+^ CXCR4^+^ cells were transduced with EasiLV vectors expressing FLAG-tagged CD8 (negative control), MX2_26–715_ (Short), wild-type MX2 (WT), or a series of MX2 constructs that were mutated at predicted stalk oligomerization interfaces 1, 2, and 3 or the BSE-hinge (H) region or that were deleted of the L4 loop (ΔL4). Cells were treated with doxycycline (0.5 μg/ml) for ∼72 h prior to challenge with an HIV-1-based lentiviral vector expressing GFP (HIV-1/GFP) at an MOI of 0.2. After ∼48 h postchallenge, HIV-1/GFP transduction efficiency was assessed by flow cytometry. Mean percentages of transduced cells from three independent experiments with standard deviations are shown. (Lower) Immunoblot analysis of parallel samples from the upper panel. Levels of FLAG-tagged proteins were determined, and Hsp90 was included as a loading control.

The ability of these MX2 stalk mutants to inhibit HIV-1 infection was tested (Fig. 2). Wild-type and mutated FLAG-tagged constructs were expressed in U87-MG CD4^+^ CXCR4^+^ cells using the doxycycline-inducible EasiLV lentiviral vector system (3), as confirmed by immunoblot analysis of cell lysates (Fig. 2, lower). Transduced cells subsequently were challenged with an HIV-1-based lentiviral vector (HIV-1/GFP), and transduction efficiency was assessed 48 h postchallenge by flow cytometry. In agreement with previous studies, wild-type human MX2 inhibited HIV-1 infection by ∼90% relative to that of the CD8 negative control, whereas the sole expression of the MX2_26–715_ short isoform had no antiviral effect (Fig. 2, upper) (3, 20, 23). Dimer interface mutants M574D, Y651D, and F647D all exhibited an essentially complete loss of antiviral activity, while V578D exhibited a partial loss of activity (∼80% inhibition relative to that of the CD8-negative control). Mutations at stalk interface 1 (M666D and I423D) had no effect on antiviral activity (>90% inhibition), whereas mutations at interface 3 yielded variable results, with R455D retaining modest activity (∼50% inhibition) but YRGK_487_-AAAA_490_ and G439D both retaining full activity. BSE-stalk hinge region mutants E681A and R689A also exhibited a significant reduction in antiviral activity (∼55 to 65% inhibition), and deletion of the L4 loop moderately affected function (ΔL4; ∼80% inhibition). The dimer interface mutants M574D, Y651D, and F647D were consistently expressed at slightly lower levels than V578D (Fig. 2, lower), perhaps contributing to the severity of the impairment to antiviral activity observed for these mutants. However, all other mutant proteins tested exhibited similar or higher expression levels than wild-type MX2.

### Characterizing the oligomerization state of MX2 stalk mutants.

**FIG 3:**
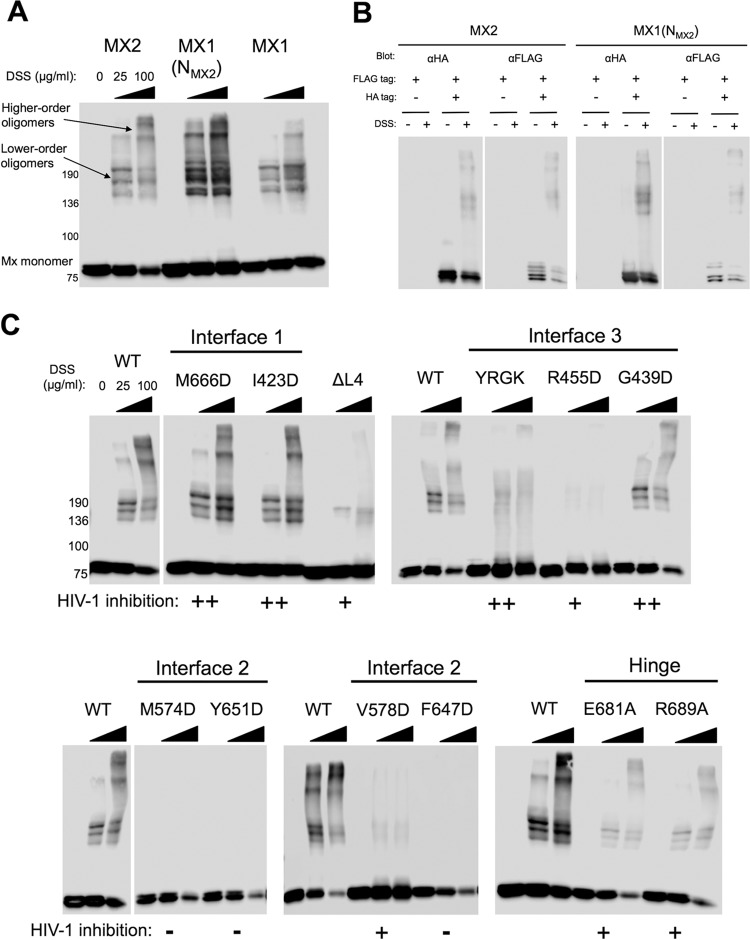
Chemical cross-linking reveals oligomerization state of MX2 stalk mutants. (A) U87-MG CD4^+^ CXCR4^+^ cells were transduced with EasiLV vectors expressing FLAG-tagged MX1, MX2, or MX1(N_MX2_), and protein expression was induced by the addition of doxycycline for ∼72 h. Cells were harvested and lysed, and disuccinimidyl suberate (DSS) was added to lysates at a concentration of 25 or 100 μg/ml (0 indicates DMSO-only control). After 1 h the reaction was quenched, and FLAG-tagged protein was resolved by SDS-PAGE and detected by immunoblotting. (B) Cross-linking followed by immunoprecipitation. 293T cells were cotransfected with FLAG-tagged MX2 WT or MX1(N_MX2_) WT and HA-tagged MX2 WT or MX1(N_MX2_) WT. After ∼30 h cells were lysed, protein cross-linked with DSS, and HA-tagged protein immunoprecipitated with anti-HA antibody. Cells transfected with FLAG-tagged constructs alone were included as a negative control. Immunoblots of immunoprecipitated protein were probed with both anti-HA and anti-FLAG antibodies. (C) Cross-linking profiles of FLAG-tagged MX2 stalk mutants. U87-MG CD4^+^ CXCR4^+^ cells were transduced, the expression of FLAG-tagged constructs was induced, and DSS cross-linking was performed as described for panel A. FLAG-tagged protein was resolved by SDS-PAGE and detected by immunoblotting. MX2 WT was included on each membrane to enable the direct comparison of the mutants to the wild type. The HIV-1 inhibition phenotype of each mutant from Fig. 2 is indicated (calculated as percent inhibition relative to that of the CD8 control); −, <50%; +, 50 to 85%; ++, >85%.

To investigate further the relationship between MX2 oligomerization and antiviral activity, the oligomerization states of MX2 stalk mutants were addressed. Chemical cross-linking of protein from mammalian cell extracts with disuccinimidyl suberate (DSS), a noncleavable, amine-reactive N-hydroxysuccinimide (NHS) ester, enabled the identification of lower-order and higher-order oligomers for wild-type MX1 and MX2, chimeric MX1(N_MX2_), and the MX2 stalk variants (Fig. 3). U87-MG CD4^+^ CXCR4^+^ cells were transduced with EasiLV vectors expressing FLAG-tagged constructs, and expression was induced with doxycycline. DSS cross-linking of cell lysates revealed a similar concentration-dependent distribution of lower- and higher-order oligomers for MX1, MX2, and MX1(N_MX2_) (Fig. 3A). Monomeric proteins were observed at ∼80-kDa (76-kDa MX1 and 78-kDa and 76-kDa MX2 isoforms), with further species migration between the 136-kDa and 190-kDa markers corresponding to lower-order oligomers. The approximate molecular masses of these species are broadly consistent with those of dimers and trimers, respectively, although this has not been formally proven. The presence of more than two discrete species within this region of the gel indicates the presence of additional cross-linked (as yet unidentified) MX binding partners. Discrete high-molecular-mass bands most likely corresponding to tetramers and/or higher-order oligomers also can be identified for MX1, MX2, and MX1(N_MX2_), with an increase in abundance of these species observed with the higher DSS concentration (Fig. 3A).

As a control to confirm that slower-migrating cross-linked complexes represented adducts containing multiple MX protein molecules rather than complexes with other cellular proteins, FLAG-tagged and HA-tagged wild-type MX2 or chimeric MX1(N_MX2_) were coexpressed in 293T cells (data not shown), lysates cross-linked, and HA-tagged protein immunoprecipitated (Fig. 3B). As shown by subsequent immunoblotting, complexes containing FLAG-tagged MX proteins were readily isolated with the HA-specific antibody, comparable distributions of the higher-molecular-mass cross-linked species were observed when probing for HA- or FLAG-tagged proteins, and the banding patterns were similar to that observed previously (Fig. 3A). Together, these data show that our cross-linking approach measures the oligomerization of MX proteins, as opposed to the formation of complexes with additional cellular proteins.

MX2 stalk mutants varied considerably in their ability to oligomerize (Fig. 3C), but in general lower-order oligomer formation corresponded with antiviral activity. Dimer interface (interface 2) mutants M574D, Y651D, and F647D lost the ability to form oligomers, with monomeric protein being the only species detected in this assay. This result is consistent with these residues being important for maintaining the dimer interface, supporting recent structural studies (6). Importantly, each of these three mutants exhibited a complete loss of antiviral activity (Fig. 2). V578D, also located at the predicted dimer interface, formed lower-order oligomers inefficiently with undetectable higher-order oligomerization (Fig. 3C) and had partial antiviral activity (Fig. 2).

In contrast, stalk interface 1 mutants M666D and I423D did not affect MX2 oligomerization, with a distribution and abundance of lower- and higher-order oligomeric species comparable to that of wild-type MX2 (Fig. 3C). These data call into question the physiological relevance of predicted stalk interface 1, and certainly these residues are not required either for oligomerization or the antiviral activity of MX2 (Fig. 2). Stalk interface 3 mutant G439D had no oligomerization defect, while L2 loop mutant YRGK_487_-AAAA_490_ formed lower-order oligomers but exhibited significantly impaired higher-order oligomerization (Fig. 3C). Both mutants, however, retained full anti-HIV-1 activity (Fig. 2). R455D, however, exhibited barely detectable lower-order oligomerization, no detectable higher-order oligomerization (Fig. 3C), and significantly reduced antiviral activity (Fig. 2). The BSE hinge region mutants E681A and R689A both formed lower-order and higher-order oligomers, albeit at reduced abundance compared to that of wild-type MX2 (Fig. 3C). Interestingly, both mutants demonstrated only modest antiviral activity (Fig. 2), perhaps due to the reduction in overall efficiency of oligomerization. Deletion of the L4 loop also incurred a significant oligomerization defect, with lower-order species barely being detected. Taken together, the infectivity and cross-linking data presented thus far imply a strict requirement for efficient lower-order oligomerization for full antiviral activity of MX2.

**FIG 4:**
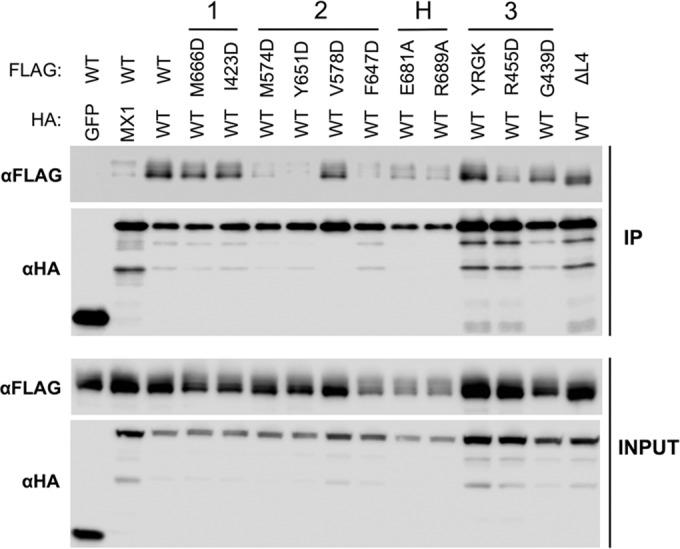
Coimmunoprecipitation of MX2 stalk mutants with wild-type MX2. 293T cells were cotransfected with HA-tagged MX2 WT and FLAG-tagged MX2 WT or stalk mutants. Cells were lysed and HA-tagged protein immunoprecipitated with anti-HA antibody. The cotransfection of HA-tagged GFP or MX1 with FLAG-tagged MX2 WT also were included as negative controls. (Upper) Immunoblots of immunoprecipitated protein (IP) were probed with anti-FLAG or anti-HA antibodies. (Lower) Immunoblots of cell lysate prior to immunoprecipitation (INPUT) were probed with anti-FLAG or anti-HA antibody as a control for protein expression.

To validate our observations regarding oligomerization of MX2 via an alternative approach, coimmunoprecipitation studies were performed with HA-tagged wild-type MX2 and FLAG-tagged MX2 stalk mutants expressed in 293T cells, HA-specific immunoprecipitation, and detection of associated FLAG-tagged proteins (Fig. 4). The assessment of coimmunoprecipitation efficiency between wild-type and mutant proteins parallels the approach previously used in the context of MX1 (10). HA-tagged GFP was included as a negative control, and HA-tagged wild-type MX1 also was included, since MX1 and MX2 do not significantly colocalize and are not believed to form hetero-oligomers (27). All HA-tagged and FLAG-tagged constructs were well expressed (Fig. 4, lower), and HA-tagged proteins were efficiently immunoprecipitated in all samples (Fig. 4, upper). As expected, FLAG-tagged wild-type MX2 was efficiently coimmunoprecipitated with HA-tagged wild-type MX2 but not with the GFP-HA control, and only a very weak interaction was observed between MX1 and MX2 (Fig. 4, upper).

In concordance with our cross-linking data, dimer interface mutants M574D, Y651D, and F647D exhibited only very weak interactions with wild-type MX2 (comparable to that observed between MX1 and MX2), while stronger interactions were observed with V578D. BSE hinge mutants E681A and R689A and stalk interface 3 mutant R455D, all of which showed reduced antiviral activity (Fig. 2), exhibited weak coimmunoprecipitation with wild-type MX2 (Fig. 4). Stalk interface 1 mutants M666D and I423D and interface 3 mutant G392D coprecipitated with an efficiency comparable to that of wild-type MX2, again correlating with the cross-linking data and their full antiviral activity (Fig. 2). The L2 loop mutant YRGK_487_-AAAA_490_ also exhibited efficient coimmunoprecipitation (Fig. 4) despite a clear defect in higher-order oligomerization (Fig. 3). Since YRGK_487_-AAAA_490_ also retained full antiviral activity, this result implies that higher-order oligomerization is dispensable for MX2 function, provided that lower-order oligomerization is sufficiently robust.

### The dimer interface is important for anti-HIV-1 activity of MX1(N_MX2_).

We next sought to determine whether oligomerization also is required for the anti-HIV-1 activity of chimeric MX1(N_MX2_). MX1(N_MX2_), a fusion of residues 1 to 91 of MX2 and 44 to 662 of MX1, which therefore replaces the native N terminus of MX1 with the extended N-terminal region of MX2, can inhibit HIV-1 with potency comparable to that of wild-type MX2 (23). MX1(N_MX2_) recapitulates many of the features associated with MX2-mediated inhibition: infection is blocked prior to nuclear cDNA import, GTPase activity is dispensable for function, and viral substrate specificity is comparable (23). In this study, the biochemical characterization of mutated proteins carrying previously described MX1 oligomerization deficiencies (10, 16) was exploited to investigate the role of MX1(N_MX2_) oligomerization in anti-HIV-1 activity. Mutations in the stalk region, BSE hinge region, and L4 loop previously shown to disrupt MX1 oligomerization (Table 1) were introduced in the context of chimeric MX1(N_MX2_). Note that, for ease of reference to previous studies, residue numbers assigned to these mutants represent the position in the native MX1 sequence.

**FIG 5:**
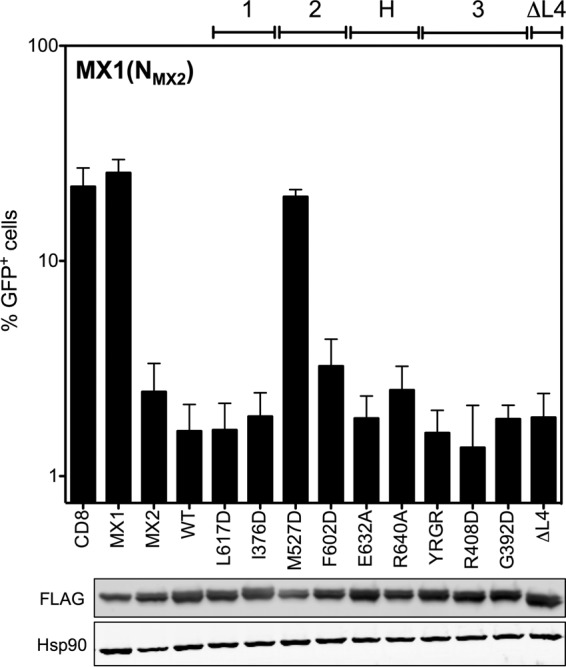
Point mutation at the dimer interface abrogates anti-HIV-1 activity of MX1(N_MX2_). (Upper) U87-MG CD4^+^ CXCR4^+^ cells were transduced with EasiLV vectors expressing FLAG-tagged CD8, MX1, MX2, and MX1(N_MX2_) WT or a series of MX1(N_MX2_) variants with mutations at predicted stalk interfaces 1, 2, and 3 and the BSE-hinge (H) region or with a deletion of the L4 loop (ΔL4). After doxycycline treatment, cells were challenged with the HIV/GFP lentiviral vector, and transduction efficiency was assessed as described in the legend to Fig. 2. Mean percentages of transduced cells from three independent experiments with standard deviations are shown. (Lower) Immunoblot analysis of parallel samples from the upper panel. Levels of FLAG-tagged proteins were determined, and Hsp90 was included as a loading control.

The ability of FLAG-tagged mutant constructs to inhibit HIV-1/GFP vector infection was tested and compared to that of wild-type MX1(N_MX2_) in a series of experiments performed in the same way as those described for Fig. 2. All FLAG-tagged constructs were well expressed in cell lysates (Fig. 5, lower). Note that chimeric MX1(N_MX2_) also exists as two isoforms due to the alternative initiation codon within the N terminus of MX2. As previously described (3, 23), MX1(N_MX2_) expression inhibited HIV-1 transduction by over 90%, at least equivalent to the inhibition conferred by MX2, while MX1 had no antiviral activity against HIV-1 (Fig. 5, upper). Strikingly, the only mutant protein that incurred any significant reduction in antiviral activity was the dimer interface mutant M527D, which exhibited a complete loss of activity.

### Characterizing the oligomerization state of MX1(N_MX2_) stalk mutants.

**FIG 6:**
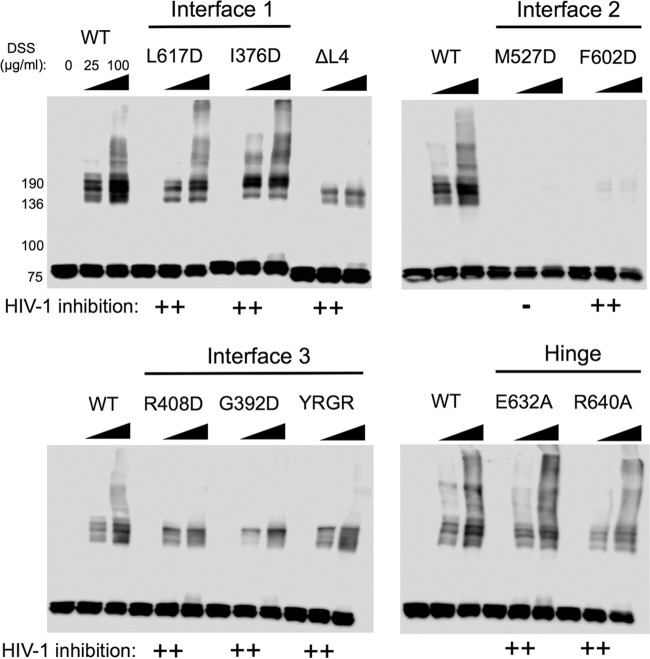
Chemical cross-linking of MX1(N_MX2_) mutants. U87-MG CD4^+^ CXCR4^+^ cells were transduced with EasiLV vectors expressing FLAG-tagged MX1(N_MX2_) WT or MX1(N_MX2_) stalk mutants, and DSS cross-linking was performed as described in the legend to Fig. 3. FLAG-tagged protein was resolved by SDS-PAGE and detected by immunoblotting. The HIV-1 inhibition phenotype of each mutant from Fig. 5 is indicated (calculated as percent inhibition relative to that of the CD8 control); −, <50%; +, 50 to 85%; ++, >85%.

The oligomerization state of FLAG-tagged MX1(N_MX2_) stalk mutants was assessed by protein cross-linking as shown in Fig. 3. The dimer interface mutant M527D, the only MX1(N_MX2_) stalk mutant analyzed here to lose antiviral activity (Fig. 5), also was the only mutant for which monomeric protein was the only species detected (Fig. 6). While the other dimer interface mutant, F602D, also incurred a significant oligomerization defect, faint lower-order oligomeric species still were detectable, and this presumably was sufficient to support antiviral function (Fig. 5). All other mutants tested exhibited an efficiency of lower-oligomer formation comparable to that of wild-type MX1(N_MX2_) (Fig. 6). Stalk interface 3 mutants and the L4 loop deletion exhibited no or barely detectable higher-order oligomerization, in agreement with a previous biochemical characterization of MX1 (10). The observation that these mutants, together with F602D, retain full antiviral activity provides strong evidence that higher-order oligomerization is not required for the inhibition of HIV-1 infection by MX1(N_MX2_). Stalk interface 1 mutants and BSE hinge mutants in the context of MX1(N_MX2_) incurred no observable oligomerization defect in this assay.

**FIG 7:**
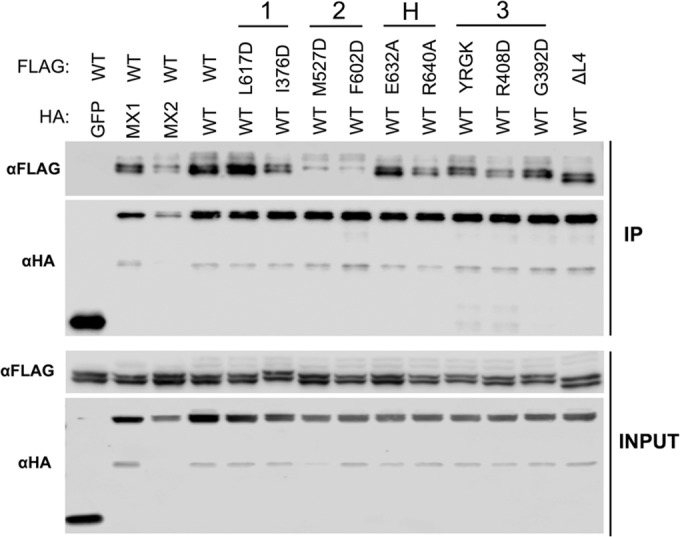
Coimmunoprecipitation of MX1(N_MX2_) stalk mutants with wild-type MX1(N_MX2_). 293T cells were cotransfected with HA-tagged MX1(N_MX2_) WT and FLAG-tagged MX1(N_MX2_) WT or stalk mutants. Immunoprecipitation of HA-tagged protein with anti-HA antibody was performed as described in the legend to Fig. 4. (Upper) Immunoblots of immunoprecipitated protein (IP) were probed with anti-FLAG or anti-HA antibodies. (Lower) Immunoblots of cell lysate prior to immunoprecipitation (INPUT) were probed with anti-FLAG or anti-HA antibodies as a control for protein expression.

Coimmunoprecipitation studies also were performed with HA-tagged wild-type MX1(N_MX2_) and FLAG-tagged MX1(N_MX2_) stalk mutants, similar to those described for Fig. 4. Again, all HA-tagged and FLAG-tagged constructs were well expressed in transfected cells (Fig. 7, lower). As expected, FLAG-tagged wild-type MX1(N_MX2_) failed to coprecipitate with the GFP-HA negative control, but efficient coimmunoprecipitation was seen with the HA-tagged wild-type MX1(N_MX2_) positive control (Fig. 7, upper). Robust coimmunoprecipitation also was observed between MX1 and MX1(N_MX2_). A far weaker interaction was observed between MX2 and MX1(N_MX2_), but interestingly, the longer isoform of MX1(N_MX2_) coprecipitated more efficiently than the short isoform, suggesting some role for the MX2 N terminus in self-assembly or protein complex formation. In concordance with the cross-linking data, each of the MX1(N_MX2_) stalk and BSE hinge mutants tested coprecipitated efficiently with wild-type MX1(N_MX2_), with the exception of the dimer interface mutants M527D and F602D. No detectable interaction was observed with the short isoforms of either mutant, while a weak interaction was observed with the long isoform.

## DISCUSSION

The ability to form structured higher-order oligomers is a common feature of dynamin-like GTPases (21), and, in the case of human MX1, higher-order oligomerization is critical for antiviral activity against FLUAV (10). In the current study, we investigated the importance of oligomerization for the antiviral activity of MX2, a closely related family member and potent inhibitor of HIV-1 infection (3–5). Previous biochemical characterization of MX1 oligomerization (10, 16) and recent structural data obtained for N-truncated MX2 (6) guided the construction of oligomerization-defective mutant proteins in the context of both MX2 and chimeric MX1(N_MX2_).

Our data demonstrate a clear requirement for the oligomerization of MX2 for antiviral activity against HIV-1 (Table 2), in agreement with previous reports (6, 31). Monomeric mutants (defined here as oligomerization being undetectable by protein cross-linking) all incurred a complete loss of antiviral activity (Fig. 2 and 3). These included M574D, Y651D, and F647D, all with substitutions at the MX2 dimer interface. Our study also reveals a relationship between the extent or efficiency of lower-order oligomerization and the potency of antiviral activity. MX2 mutants exhibiting reduced but still measurable lower-order oligomerization by cross-linking, including V578D at the dimer interface, R455D at stalk interface 3, and the L4 loop deletion mutant, all displayed partial antiviral phenotypes. Although the coimmunoprecipitation data broadly concurred with oligomerization phenotypes determined by cross-linking, efficient coimmunoprecipitation was observed for some mutants with clearly reduced antiviral activity, and a weak but detectable coimmunoprecipitation was noted with dimer interface mutants shown to be monomeric by cross-linking (Fig. 4). Since coimmunoprecipitation was performed with cell lysates and is not dependent upon the proximity of interacting proteins (the distance constraint for DSS protein cross-linking has been estimated to be 26 to 30 Å between alpha carbon atoms of cross-linked lysine residues [32]), it is possible that some coimmunoprecipitation reflects the isolation of MX protein-containing multiprotein complexes rather than direct MX-MX interactions. Alternatively, the stability of interactions between wild-type and mutant proteins, when assessed by coimmunoprecipitation, may appear greater than the stability of homotypic mutant protein interactions detected by protein cross-linking.

**TABLE 2:** Oligomerization and HIV-1 inhibition data for MX2 and MX1(N_MX2_) mutants^*a*^

Mutant	Site	Inhibition of HIV-1^*b*^	Oligomerization by cross-linking	Coimmunoprecipitation
MX2				
WT		++	Higher order	Yes
M666D	Stalk 1	++	Higher order	Yes
I423D	Stalk 1	++	Higher order	Yes
**M574D**	**Stalk 2**	**−**	**Monomeric**	**Very weak**
**Y651D**	**Stalk 2**	**−**	**Monomeric**	**Very weak**
V578D	Stalk 2	+	Limited oligomerization	Yes
**F647D**	**Stalk 2**	**−**	**Monomeric**	**Very weak**
E681A	Hinge	+	Higher order	Weak
R689A	Hinge	+	Higher order	Weak
YRGK-AAAA_487–490_	Stalk 3	++	Lower order	Yes
R455D	Stalk 3	+	Limited oligomerization	Weak
G439D	Stalk 3	++	Higher order	Yes
ΔL4	L4 loop	+	Lower order	Yes
MX1(N_MX2_)				
WT		++	Higher order	Yes
L617D	Stalk 1	++	Higher order	Yes
I376D	Stalk 1	++	Higher order	Yes
**M527D**	**Stalk 2**	**−**	**Monomeric**	**Weak**
F602D	Stalk 2	++	Limited oligomerization	Weak
E632A	Hinge	++	Higher order	Yes
R640A	Hinge	++	Higher order	Yes
YRGR-AAAA_440–443_	Stalk 3	++	Higher order	Yes
R408D	Stalk 3	++	Lower order	Yes
G392D	Stalk 3	++	Lower order	Yes
ΔL4	L4 loop	++	Lower order	Yes

a Summary of HIV-1 inhibition and protein oligomerization data shown in Fig. 2 to 7. The site, antiviral activity against HIV-1, oligomerization phenotype by protein cross-linking, and coimmunoprecipitation efficiency with WT or mutant proteins described in this study are shown.

b The HIV-1 inhibition phenotype indicates percent inhibition calculated relative to that of the CD8 control (from Fig. 2 and 5): −, 0 to 50%; +, 50 to 85%; ++, 85 to 100%. Mutants exhibiting a complete loss of antiviral activity are highlighted in boldface.

We observed no role for putative stalk interface 1 in either oligomerization or antiviral function of MX2, arguing against its biological relevance. Two previous studies, with the same M666D and I423D mutants, also have concluded that this purported interface is not required for antiviral activity (6, 31), although the lack of an effect on oligomerization is in agreement with one study (31) but not the other (6). The latter study was performed with recombinant, maltose-binding protein (MBP)-tagged protein expressed in Escherichia coli, which has the potential to behave differently than proteins expressed in mammalian cells. However, since our study assessed only single point mutants at interface 1, we cannot exclude the possibility that the mutations introduced here were insufficient to disrupt the interface.

The HIV-1 inhibition phenotypes observed for stalk interface 3 mutants R455D and G439D and the L4 loop deletion were in agreement with those observed previously (25), but here we show that antiviral activity correlated with the relative ability of these mutants to form lower-order oligomers. Here, we also demonstrated a role for the BSE hinge region in the antiviral activity of MX2, with mutants E681A and R689A being significantly impaired for HIV-1 inhibition, although a previous study found no effect of the E681A mutation on function (6). The impact of these mutations on the oligomerization of MX2 had not been tested previously, but here we show by protein cross-linking and coimmunoprecipitation that both mutations incur a moderate oligomerization defect that likely explains their functional impairment. That mutant R689A retained any antiviral activity stands in contrast to corresponding mutant R640A in MX1, which lost inhibitory function (16). Since the BSE hinge region has been ascribed a role in conformational coupling between the GTPase domain and the stalk, its lesser importance for MX2 activity may have been anticipated given the GTPase independence of this protein (3, 4).

Extending these studies, we also demonstrate the requirement for lower-order oligomerization in the context of the MX1(N_MX2_) chimera, implying that this attribute enables the MX2 N-terminal domain to mediate antiviral activity. Dimer interface mutant M527D, which failed to form any detectable oligomeric species by protein cross-linking, exhibited a complete loss of antiviral activity (Fig. 5 and 6). However, mutant F602D, which is also markedly defective for oligomerization but still showed very faint lower-order cross-linking, was fully antiviral. Perhaps HIV-1 is more sensitive to inhibition by MX1(N_MX2_) than MX2, such that only a small proportion of the active form is required. Indeed, the HIV-1 suppression phenotype observed upon the expression of MX1(N_MX2_) typically is stronger than that for MX2 (23).

The data presented here corroborate previous observations using constructs in which the amino-terminal 91 residues of MX2 were fused to monomeric, dimeric, or trimeric versions of the yeast GCN4 leucine zipper domain (20). Dimeric and trimeric constructs retained ∼80% antiviral activity, while the monomeric construct was not antiviral, implying that the dimerization of the MX2 N terminus is sufficient for antiviral activity to be elicited (20). Extending this observation, we now conclude that higher-order oligomerization is dispensable for full antiviral activity, particularly in the context of MX1(N_MX2_). Dimer interface mutant F602D, stalk interface 3 mutants R408D, G392D, and YRGR_440_-AAAA_443_, and the L4 loop deletion all exhibited no (or barely) detectable higher-order oligomerization yet retained full antiviral activity in this context. The dependence of MX2 activity upon dimerization is reminiscent of fusions between the murine leukemia virus restriction factor Fv1 and cyclophilin A; these chimeric proteins also suppress infection by inhibiting viral cDNA nuclear import, perhaps indicating commonalities in mechanism (33, 34).

Notably, wild-type MX2 and MX1(N_MX2_) both exhibited profiles of lower-order and higher-order oligomeric forms by cross-linking that were similar to those of MX1 (Fig. 3A). However, and despite the similarities in structure and propensity to form higher-order oligomers, current data support the conclusion that there are a number of fundamental differences between the antiviral mechanisms of MX1 and MX2.

The mechanism underpinning the requirement for MX2 oligomerization is not presently understood. Previous studies have shown that monomeric MX2 mutants fail to bind HIV-1 capsid-nucleocapsid nanotubes *in vitro* (6, 31). However, further investigation is required to determine the precise nature and relevance of the interaction between MX2 and CA, since mutations in CA that permit viral escape from MX2-mediated inhibition do not block MX2 binding *in vitro* (7, 8). The potential involvement of additional cellular factors, as well as the importance of MX2 oligomerization for their recruitment, will require future exploration.
